# Identifying the Leadership Challenges of K-12 Public Schools During COVID-19 Disruption: A Systematic Literature Review

**DOI:** 10.3389/fpsyg.2022.875646

**Published:** 2022-03-31

**Authors:** Khalida Parveen, Phuc Quang Bao Tran, Abdulelah A. Alghamdi, Ehsan Namaziandost, Sarfraz Aslam, Tian Xiaowei

**Affiliations:** ^1^Faculty of Education, Southwest University, Chongqing, China; ^2^Faculty of Education, Umm Al-Qura University, Makkah, Saudi Arabia; ^3^University of Applied Science and Technology, Khuzestan, Ahvaz, Iran; ^4^School of Foreign Languages, Yulin University, Shaanxi, China

**Keywords:** leadership challenges, COVID-19 pandemic, educational crises, systematic literature review, school leaders

## Abstract

Globally, the COVID-19 pandemic is triggering a public health emergency and crisis on a large scale, with far-reaching effects and severe damage to all aspects of politics, economy, cultural and social life, and health. Consecutive outbreaks over the past nearly 2 years of “living with COVID-19” have forced most schools to physically close, resulting in the largest educational disruption in human history. In turbulent times of the COVID-19 crisis, school leaders are facing numerous major challenges germane to school governance and leadership. The key objective of the study is to fully explore the prospective challenges principals are encountering in public schools in times of COVID-19. To fulfill the research purpose, a systematic literature review (SLR) was carried out to investigate the leadership challenges. As a result, a total of 24 challenges were explored through SLR approach. Frequency analysis approach was initially applied to figure out the most significant challenges. Accordingly, seven challenges were found statistically significant as showing frequency ≥ 50 each. Irrevocably, the study works as a contribution to K-12 school leadership by providing guidance for current and future leaders in crisis based on practical investigation, experiences, and recommendations. Policy makers can leverage these findings to make necessary adjustments to school policy to better prepare school leaders for crisis. Additionally, the findings of the current study are believed to have profound implications for future research. These findings expand our current understanding on school leadership in time of crisis that needs further investigation. Subsequent studies can quantitatively and/or qualitatively validate these leadership challenges findings regarding a particular school context.

## Introduction

### COVID-19 and the Educational Crisis

In December 2019, initial confirmed COVID-19 cases were detected in Wuhan, China (Vlachopoulos, 2020; Huang et al., 2020a). Since the World Health Organization (WHO) declared a global pandemic in March 2020 (WHO, 2021a), subsequent variants of COVID-19 have starkly shaken the world by their deadly nature and uncontrollable speed of spread. The whole world has become the COVID-19’s playground. During the final stage of this research work, more than 200 countries and territories lay upended when the COVID-19 hit hard.

Accordingly, hundreds of millions of cases have been recorded worldwide and the price to pay for this unequal battle is the lives of millions of innocent people (Corlatean, 2020). So far, World Health Organization has presented latest data with a total of more than 230 million coronavirus patients and approximately 4.8 million deaths (WHO, 2021b). It seems that the pandemic has not yet created enough of the damage to all fields of economy, politics, society, foreign affairs, and security of countries and regions worldwide. The heat of the pandemic has not cooled down and this battle seems to show no signs of ending.

The lockdown, social distancing, and travel restriction measures introduced by the governments and widely put in place in various forms to contain the coronavirus and mitigate the loss of human are also having a direct impact on people’s lives (World Bank, 2020a). The vulnerable groups in society become prey for escalating inequality related to access to quality healthcare, job security, safety, adequate food, education, and vaccination (World Bank, 2020a). In other words, the COVID-19 pandemic and its consequences are directly depriving people of their basic human rights, of which is the right to equal access to education. Nearly 2 years of “living in the reign” of COVID-19, the major concern is the disruption in terms of education.

The interruption of education triggered by repeated school closures following the successive outbreaks during the academic years of 2020 and 2021 is unprecedented in modern education. The implementation of lockdowns, social distancing, and travel restrictions on a global scale has hit local and international educational institutions at all levels of schooling, teachers, and students to varying degrees (UNESCO, 2020; United Nations, 2020b). These quarantine measures have imposed serious constraints on all schools and created a bleak landscape for the global education community.

The COVID-19 pandemic has not spared the education of any country, both rich and poor (World Bank, 2020a). Most educational and training institutions have had to switch to remote or hybrid learning in the context of urgency, lack of preparation, limited resources, teacher shortage, unequal online educational experiences, and even digital illiteracy (UNESCO, 2020; World Bank, 2020a). Accordingly, many schools are newcomers with no prior experience in online education compounded by complete passivity in terms of the capacity of administrators and teachers, and inadequate facilities and technical infrastructure.

Significantly, school closures or resumptions overnight during COVID-19 outbreaks raise greater public concern over the safety of the school community, learning loss, the quality of instruction modalities, teaching and learning organization plans, compensation for learning loss, student progress and achievement, and safety return post-pandemic (Reimers and Schleicher, 2020; Huang et al., 2020b). The closure of schools and the rush to switch to online learning have also exposed the already-existing digital divides and socioeconomic inequalities between countries, between educational institutions, and among students (Azevedo et al., 2020).

Schools in low- and lower-middle-income countries are still struggling to sustain student learning and deliver quality remote learning programs due to limitations of facilities, technological infrastructure, funding, additional resources, and a lack of experienced academics (Khlaif and Salha, 2020). Additionally, concerns over the psycho-physiological health of schools’ stakeholders, especially young students (United Nations, 2020a), have grown as lingering inconspicuous COVID-19-related consequences can wreak havoc for long.

The COVID-19 crisis has exposed the leadership confusion in addressing the issue of “no one is left behind.” As the center of social activities and human interactions, when schools are physically shut down, millions of young students and youth miss out on the opportunities and social interactions essential for learning and developing life skills (World Economic Forum, 2020; Ewing and Cooper, 2021). It is estimated that millions of children of marginalized groups in any society are at risk of dropping out of school (UNESCO, 2021a). Sadly, even with continued education in any form, hundreds of millions of children fail to achieve the minimum proficiency in reading and writing, and numeracy (United Nations, 2020b; UNESCO, 2021a), falling behind their better-off peers due to disruption of schooling (United Nations, 2020b).

Devastatingly, the risk of not attending school during the disruptive times or in the aftermath of COVID-19 entails various dire consequences for children and youth, namely, being subjected to domestic violence, caught up in the labor market, and the risk of female children being abused and forced into early marriage (Rogers and Sabarwal, 2020; United Nations, 2020a). The longer schools are physically closed, the greater the risk that young students lose their future opportunities (United Nations, 2020b).

### Study Objectives and Research Question

In the turbulent era of the COVID-19 pandemic causing sudden disruptions to global education, school leaders worldwide are facing numerous major challenges related to school governance and leadership. As schools quickly change their operations and organizational structure to respond to COVID-19, the emergence of pre-existing and new challenges is unavoidable and can become more complicated. As school leaders worldwide are navigating challenges triggered by the reign of COVID-19, various initial research has attempted to identify the critical challenges. With a purpose of systematically synthesizing existing literature related to this topic as well as adding more insights into the leadership knowledge, we primarily conducted a systematic literature review (SLR) to investigate the critical leadership challenges facing public school principals when they are navigating the impact of COVID-19. Accordingly, we also attempted to offer a thorough discussion on the identified challenges in order of frequency significance. The following key research question was formulated as “what are critical leadership challenges confronting public school principals as identified by a systematic literature review (SLR)?”

## Literature Review

### School Leadership in Uncertain Times of COVID-19

The burgeoning COVID-19 crisis is redefining school leadership. Time is short to think twice, and school leaders need to make informed choices about the priority of their operational strategies and policies in the face of COVID-19. Accordingly, the first priorities are to keep track of all the students, ensure their physical and mental wellbeing, make sure students’ education is on track, and monitor student progress (Harris, 2020; Kaul et al., 2020). “No child left behind” should be the first and only ethos regardless of any form of learning or institutional response to COVID-19.

In these most uncertain times, school leaders struggle to maintain a balance between complying with local COVID-19 infection prevention and control protocols, soberly leading the school community through the pandemic safely, meeting local educational needs, and supporting the community materially and mentally (Kaul et al., 2020; Stone-Johnson and Miles Weiner, 2020). Leadership also needs to monitor the current response to the crisis along with thinking strategically to overcome challenges and mitigate the educational damage in the post-COVID-19 era (Rogers and Sabarwal, 2020; Zhao, 2020). It is also a time to test their schools’ crisis management team and to update their crisis management plan (Mawhinney et al., 2019).

The COVID-19 era has also tested the leadership capacity of school leaders in unprecedented ways, forcing them to make quick and critical decisions to protect the wellbeing of the academic community, maintain the continuity of the organization, and adjust the school education plan according to the direction of the governing body. This is a time when school leadership is further focused on addressing teacher and parent social and emotional concerns and engaged in more constructive dialogues with stakeholders. In turbulent times of pandemic, school leaders are forced to think fast, act fast, and fear less (Leithwood et al., 2020; Netolicky, 2020).

It is no exaggeration to say that schools have become resilient fortresses against the onslaught of the pandemic. In the face of unpredictable developments of COVID-19, resilience, flexibility, adaptability, compassion, and trust are key for school leaders to being able to live with the pandemic (Beauchamp et al., 2021; De Martino and Weiser, 2021; Longmuir, 2021; Oplatka and Crawford, 2021). School leadership and management in this context have also become necessarily synonymous with ongoing support, collective collaboration, and distributed leadership (Harris and Jones, 2020; Rogers and Sabarwal, 2020).

Additionally, local communities are a noteworthy key resource for principals, due to their host of an asset of supplementary knowledge and additional capacity (Harris and Jones, 2020). Constructing stronger connection with social groups and parents is of great benefit to assist marginalized, vulnerable populations in navigating the prevailing problems that pandemic has created intensely (Harris and Jones, 2020; Stone-Johnson and Miles Weiner, 2020). Furthermore, school leadership has turned into distributed leadership response to mobilize collective efforts as schools grow through the pandemic (Azorín et al., 2020). COVID-19 is genuinely the perfect time for school stakeholders to practice connecting, sharing and mutual understanding, particularly for principalship.

COVID-19 is not only a test of leadership resilience since the future can promise more volatility with greater impact, but also an opportunity to create better and more resilient education (UNESCO, 2020; Zhao, 2020). Owing to the emergence of COVID-19, school leaders seize this once-in-a-lifetime opportunity to re-evaluate their long-term policy and the urgency of sustainable development, transform their leadership and management models, and increase their ability to respond to crisis in the local and international context with many unforeseen changes (Harris and Jones, 2020; Zhao, 2020).

### Leadership Challenges in Turbulent COVID-19

A rigorous review of existing literature provides a universal list of challenges facing school leaders in the turbulent era of COVID-19, including continuity of learning and quality of virtual or blended education, digital divides, digital literacy and professional development, inequalities in access to education, equity gaps, school financing, cyber security for online education, safety of school reopening, and emotional and mental health.

In times of school disruptions, blended online learning is hailed as practical for its feasibility of synchronous and asynchronous modes. The real challenge here is to get students motivated and engaged in blended strategies of learning to mitigate learning loss and sustain the quality of instruction, though online or in-person. The adoption of educational technology requires a sufficient level of digital literacy that many school stakeholders of materially impoverished groups are falling behind. As well, as school leaders, the principals must have the foresight for dynamic, efficient, and operative principles in tackling matters among the host community and school staff, it is only possible by adequate and continuous professional growth and advancement (Adarkwah, 2021; Akram et al., 2021; Aslam et al., 2021).

Thanks to COVID-19, wide disparities in accessing Internet and digital devices expose themselves to the public eyes (UNICEF, 2020). During the first year of pandemic, globally, roughly 2.2 billion children and aged 25 years or less youths—two-thirds of the world’s children and young people—fail to afford a reliable Internet connectivity (UNICEF, 2020). Even in the United States, the closure of schools made millions of low-income US households with K-12 students suffer from lack of computers and high-speed Internet connection (United States Facts, 2020). To bridge the gap on the Internet and technology divides, some American principals developed initiatives that mobilize collective resources for the distribution of computers and smartphones. One Montana-based principal leading a community school on Native American reservation struggled to partner with a local Internet provider to provide an “Internet hub” in the school parking lot for their community (Kaul et al., 2020).

Furthermore, COVID-19 has deep cut into the inequalities in access to education of marginalized students (Van Lancker and Parolin, 2020), regardless of whether their residencies are in a rich or poor country. The situation is even worse in school communities that are impoverished or with frequent armed conflicts, civil wars, or even ethnic divisions. The crisis is exacerbating pre-existing educational disparities, by depriving many of the most vulnerable young students of learning opportunities (Alasuutari, 2020; United Nations, 2020a). Due to the lack of schooling, vulnerable children are increasingly subjected to physical and psychological abuse, exploitation, and trafficking (Rogers and Sabarwal, 2020).

School communities’ wellbeing and mental health have become even more urgent in times of school disruptions when COVID-19 is still raging (Harris and Jones, 2020). Accordingly, physical and mental wellbeing of school principals is of foremost value due to the sheer pressure of leading and managing on the front line struggling to ensure staff and students safety (Harris and Jones, 2020). The latest Teacher Wellbeing Index report from Education Support (2020) reveals that an overwhelming 89% of school leaders experienced the feeling of “stressed” or “very stressed” since school in September. Additionally, 59% school leaders disclosed that they are considering leaving the profession this year due to greater pressures on their health and wellbeing (Education Support, 2020). As most schools are gradually moving into “new normal” beyond the pandemic, the top priority is the wellbeing of school stakeholders to sustain the “usual” track of education (McLeod and Dulsky, 2021).

Similarly, principals are struggling financially for the improvement of school functioning as schools are operating in various learning modalities. Before COVID-19, the average education spending of primary schooling of high-income countries was 43 times higher than that of low-income countries (World Bank, 2020b). In some global corners, growth in education spending is likely to continue but at remarkably lower rates than before (World Bank, 2020b). Particularly, the forecast for high-income countries’ education spending presents a decline in tandem with overall government spending (World Bank, 2020b).

The world is adjusting to “new” life in the midst of a pandemic, and the same is true for education sector. Responding to the COVID-19 pandemic and its aftermath will be one of the biggest challenges for school leaders in this era. By being open and sharing insights with each other, school leaders are using data and latest tools to quickly adapt and navigate COVID-19 challenges. More than just a trend, this “new normal” will have lasting and powerful effects on the future of education and school leadership.

## Research Methodology

**Figure 1:**
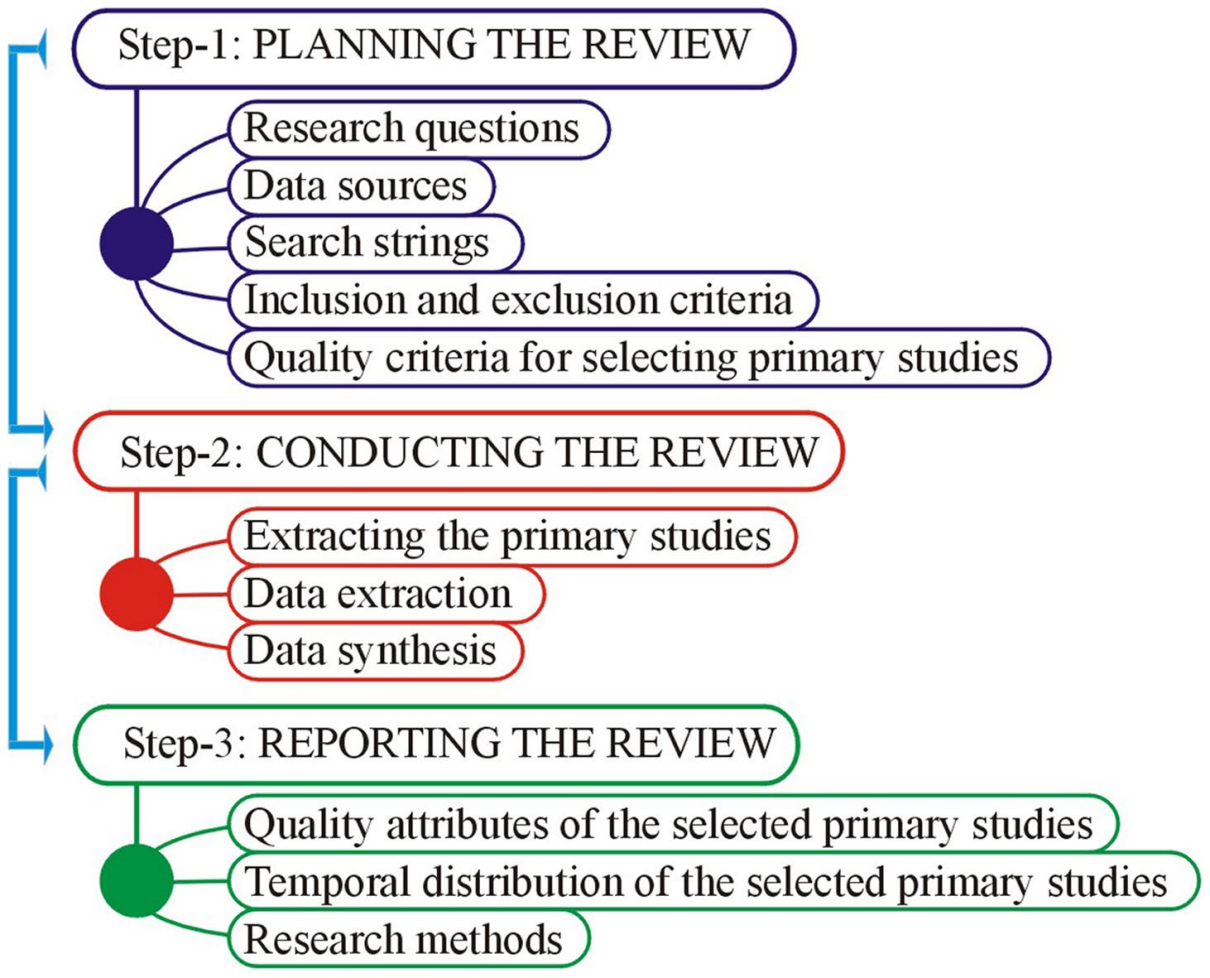
Steps of Systematic Literature Review.

To acquire the requisite objectives of the study, a Systematic Literature Review (SLR) was conducted. SLR is a significant investigation research approach to carry evidence-based research to gain a better insight into the research question or topic of interest (Davies et al., 2013; Hallinger, 2013; Siddaway et al., 2019). SLR allows researchers to collect and critically analyze multiple research studies through a systematic process with lower bias than traditional literature reviews (Akbar et al., 2019; Gümüş et al., 2021). Accordingly, SLR provides an organized approach to investigate the latest literature relevant to the study objectives. A brief discussion of the proposed method is given in the following sections. Figure 1 shows the hierarchy of steps taken in this study.

This paper carried out SLR using PRISMA guidelines. All the steps of SLR were performed by subsequent step-by-step standards recommended by Siddaway et al. (2019) and Petersen et al. (2015). The objective of current steps is to describe overview of a state-of-the-art of the concerned topic. In order to conduct the SLR study, the adopted steps are graphically shown in Figure 1 and conversed in the following sections:

### Digital Libraries Selection

Keeping in mind the previous personal experience and considering the recommendations of Chen et al. (2010) and Siddaway et al. (2019), the best suitable repositories were chosen. The objective of selecting these repositories is to bring together the most appropriate studies with high impact linked to the area of research objective. So, for getting the big population of published data and most potential information, we give priority to these seven well known digital libraries:

- Taylor and Francis^1^
- Sage Publications^2^
- Springer Link^3^
- Emerald Insight^4^
- Wiley Online Library^5^
- Academia^6^
- Google Scholar^7^

### Search String

A proper research string was used to retrieve the most related studies from these selected repositories. We made a string by using keywords and substitutes regarding our research question by keeping in mind the helpful ideas provided by Chen et al. (2010), Shafiq et al. (2020), and Astadi et al. (2022a). For the formulation of the search string, all keywords with their substitutes were connected to Boolean operator “AND” and “OR.” Furthermore, each search string was formulated according to the search structure of each database. Table 1 shows the search keywords and substitutes.

**Table 1:** Search string.

Related Topics	Search Keywords and Their Alternatives
T1 (intervention)	“Challenges” OR “barriers” OR “problems” OR “obstacles” OR “hurdles” OR “difficulties” OR “impediments” OR “hindrance”
T2 (population)	“School administration” OR “leadership” OR “school principals” OR “school management” OR “role of school heads”
T3 (institution)	“School education department” OR “SED” OR “school administration” OR “public school management” OR “principals’ role in quality management”
T4 (experimental)	“Case studies” OR “empirical studies” OR “theoretical studies” OR “quantitative surveys” OR “Qualitative surveys” OR “Mixed method surveys”
Final Search String = (T1) and (T2) and (T3) and (T4)	

### Inclusion and Exclusion Criteria

The following criteria were used to include the selected literature at an early stage (Chen et al., 2010).

- The selected paper must have been published in a conference or quality journal.
- The study must be published in English medium.
- The selected article must contain an in-depth evaluation of the challenges encountered by the heads of the schools in COVID situation.
- The study should highlight the administrative factors significant for school principals.

The following criteria were applied for the exclusion of studies (Chen et al., 2010).

- If there is no detailed description for highlighted challenges.
- Only one comprehensive study should be included in the case of two or more studies having similar findings.
- If the findings of selected studies do not construct empirical investigations.

### Study Quality Evaluation

To measure the quality of selected papers, the guidelines used are recommended by Davies et al. (2013) and Petersen et al. (2015). The quality of the selected articles entails the significance of the selected literature.

The following checklist was developed to assess the quality of the selected literature:

- Whether the research method used in study follows the requirements of the research questions?
- Whether the selected literature describes any aspect of school leadership?
- Whether the selected literature describes the school administrative strategies and its application in education sector?
- Are the provided facts relevant to school leadership in education sector?
- Do the results of the studies relate to the validation of the research questions?

Accordingly, the score “1” is administered to a study if it provides the satisfactory answer for the above listed research questions. A score “0.5” is given if the selected article provides answer to the questions of checklist to some extent and if a selected article fails to provide the answer to any checklist question, it is given a score of 0. The detailed list of selected primary studies along with showing their Quality Evaluation (QE) scores is placed at Supplementary Data Sheet 1.

### Final Selection of Primary Studies

**Figure 2:**
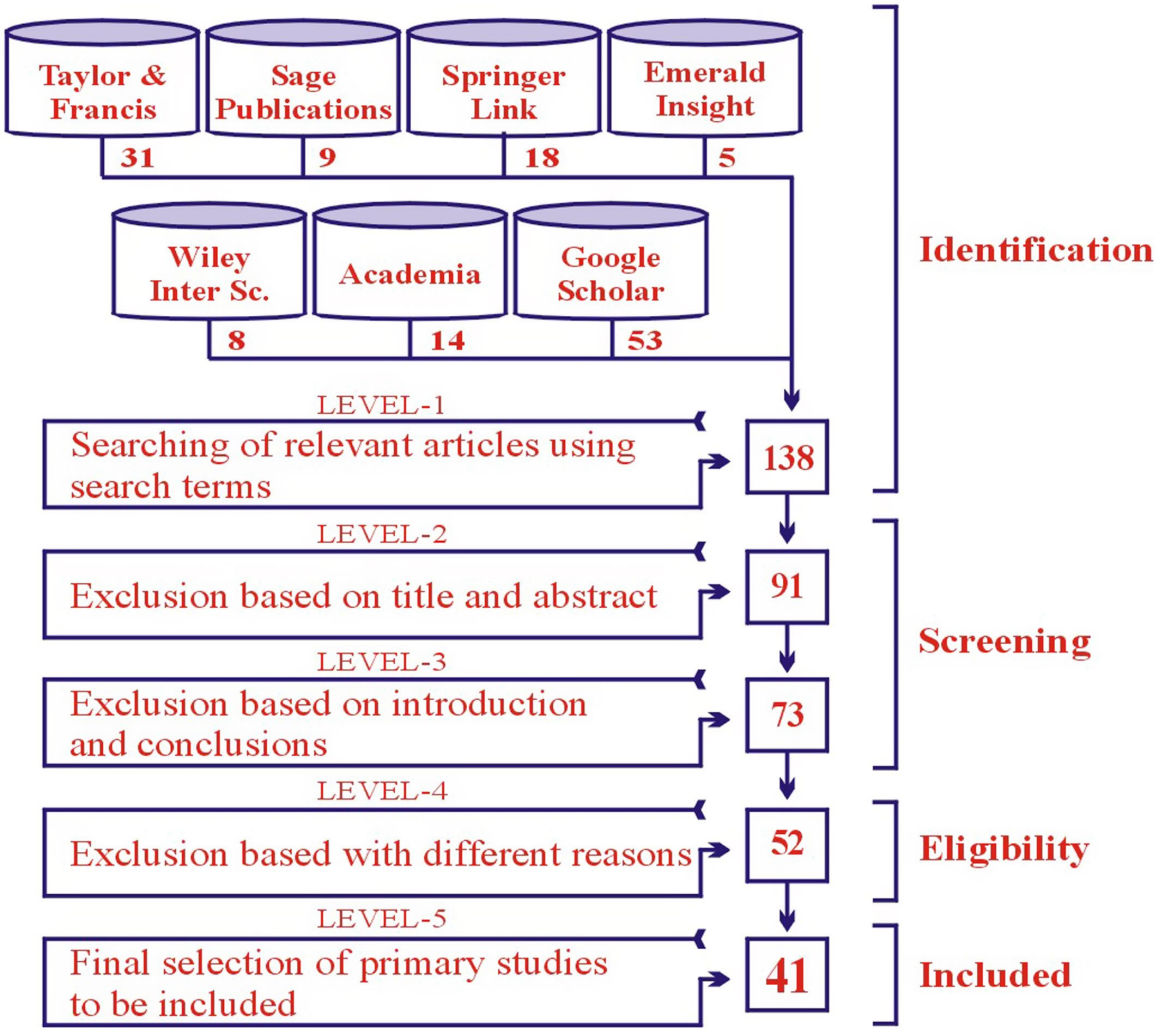
Selection of primary studies through PRISMA guidelines.

The selected primary studies were more refined by using the standards suggested by Chen et al. (2010) and Antony et al. (2012). All the phases of the tollgate approach proposed by Sniedzins (2013) were vigilantly carried out, to make better the final studies for the process of data extraction; as a result, the obtained outcomes are shown in PRISMA diagram, i.e., Figure 2.

Primarily, a total of 138 articles were chosen from the mentioned-above digital libraries by using search strings, and by applying inclusion and exclusion criteria. Finally, a total of 41 primary studies were selected to address the research question of the study. According to Lumban Gaol (2021) and Astadi et al. (2022a), the number of articles below 50 is appropriate for the literature review process. Total 26 articles from Scopus, four from WoS journals, and 11 from peer review international journals were selected (Supplementary Data Sheet 1). The QE criteria were applied to check the quality of the selected studies.

### Data Extraction and Synthesis

Out of the 41 final selected papers, data were extricated. A 4-member team was deliberately formed for the purpose of data extraction. During the process, prospective statements, main themes, and identified administrative challenges encountered by the principals during COVID-19 were noted down and imported into Microsoft Excel sheet. Primarily, 36 administrative challenge statements were highlighted and recorded into another Microsoft Excel sheet. Moreover, after consulting to research experts and team members, 36 challenges were finally merged into 24 basic prospective challenges.

After the accomplishment of the data extraction procedure, an inter-rater reliability test was administered to perform the elimination of the inter-person biasness. By following the practice of Shafiq et al. (2020), five external reviewers were requested to acknowledge their involvement for testing the selected primary studies. However, three of them were agreed to participate. One external reviewer is Associate Professor at Neijiang Normal University, Sichuan (China). Other two experts were postdoctoral research fellows from Southwest University (China). Initially, at phase 1 of the tollgate approach (Afzal et al., 2016), 15 studies were randomly selected by the external reviewers; afterward, all the stages of the Systematic Literature Review were carried out. A non-parametric Kendall’s coefficient of concordance (W) was determined in order to check the inter-rater agreement among the reviewers. To show the disagreement, the value of W = 0 is used and to show acceptance, W = 1 is denoted. The results of the inter-rater reliability test for the 15 randomly selected studies indicated that W = 0.84 (*p* = 0.0013), which showed the signed agreement between the authors and the external reviewers. The code used to perform Kendall’s coefficient of concordance is placed at Supplementary Data Sheet 2.

### Review Reporting

#### Quality Evaluation of the Selected Articles

Based on five QE questions, the QE scores for each primary study were analyzed. Accordingly, most of the primary studies achieved QE scores ≥75% after applying the QE criteria. Therefore, the achieved scores denoted that the selected primary studies are satisfactorily important to address the research question of this study. Moreover, given the selection of primary studies, a QE score of 40% as the threshold is used.

#### Temporal Distribution and Analysis of Research Method Basis

**Figure 3:**
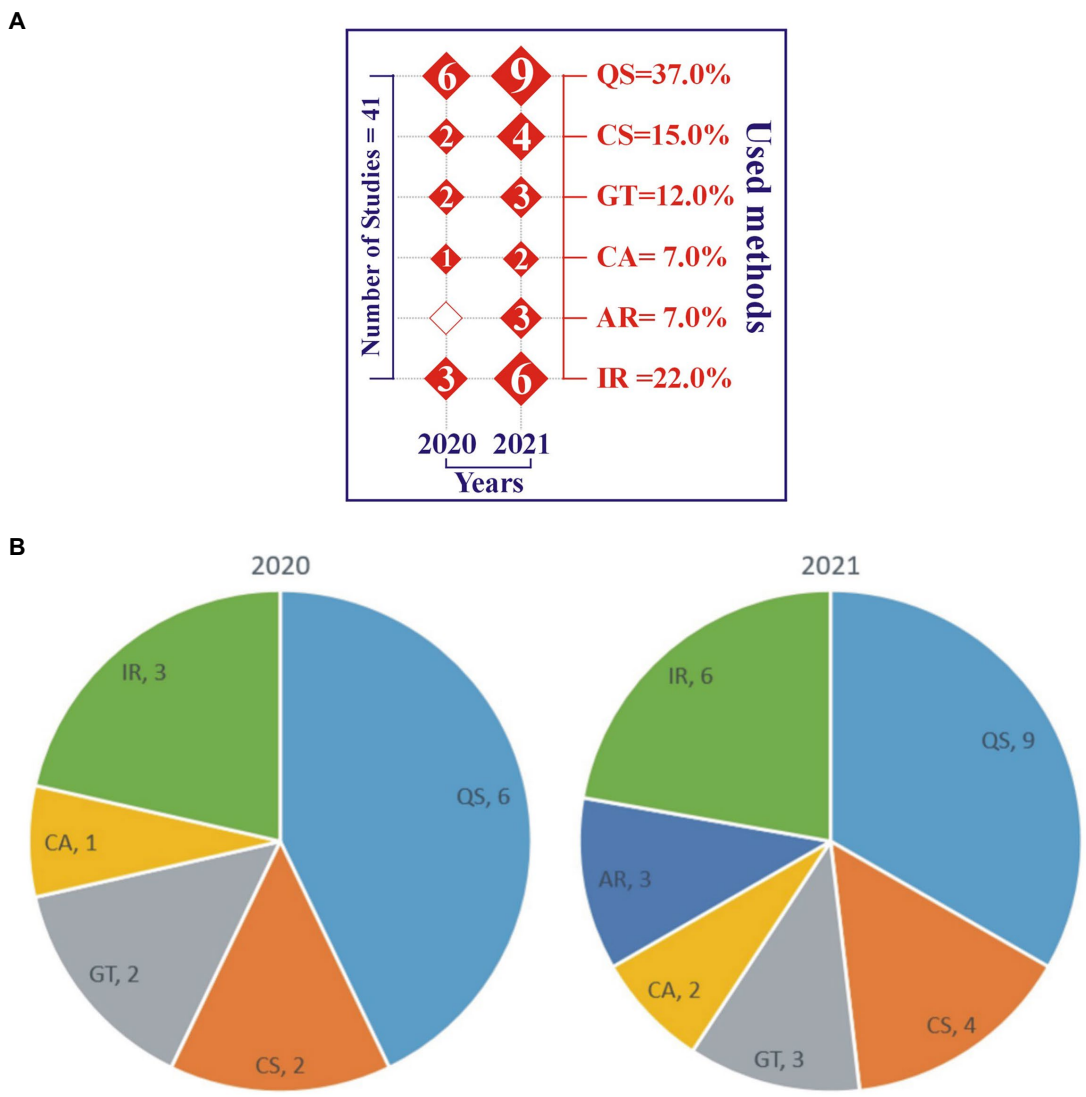
**(A)** Temporal and research methodologies-based distribution. **(B)** Temporal and research methodologies-based distribution chart.

Primary studies are chosen from the period of 2020 to 2021 (Figure 3). Thus, 34% of publications are from 2020 and 66% of publications are from 2021, respectively. Hence, there is 32% increase in the publication of articles about the school administration and leadership in times of COVID-19 situation as competed with the previous year. The ascending publication number specifies the significance of administration and leadership in the school education department and educational research.

In Figures 3A,B, the percentage of research approaches adopted in the primary studies are questionnaire survey (QS) 37.0%, case study (CS) 15.0%, grounded theory (GT) 12.0%, content analysis (CA) 7.0%, action research (AR) 7.0%, and interviews (IR) 22.0%. It is clearly shown by the analysis that questionnaire survey approach with 37.0% is mostly used as the major research methodology in the selected primary studies, and interviews with 22.0% are affirmed as the second most adopted research method in the leadership challenges investigations (Figures 3A,B).

## Results and Discussions

The primary objective of the study was to identify critical leadership challenges during the COVID-19 pandemic. We employed Systematic Literature Review to place these challenges confronting school principals in order of frequency significance. The complete list of primary selected studies is provided at Supplementary Data Sheet 1.

**Figure 4:**
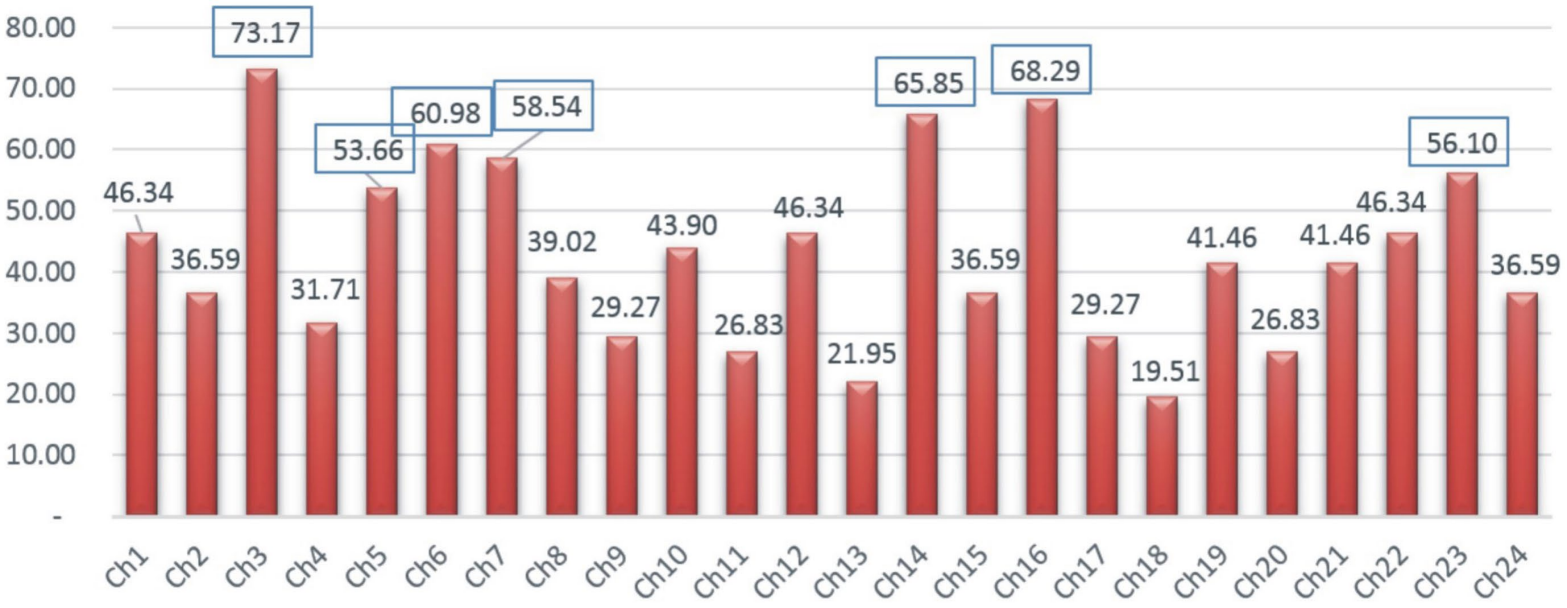
Frequency analysis of identified challenges.

In order to address the research objective, the identified challenges along with the frequency and percentage are summarized in Table 2 and Figure 4. The challenges with greater significance having frequency ≥ 50 are determined as critical. This is the same criterion adopted in other studies, such as Shafiq et al. (2020) and Akbar et al. (2020). Based on this criterion, the following challenging factors were identified as critical and discussed.

**Table 2:** List of the investigated challenges.

Sr. #	Investigated challenges	Frq. (*N* = 41)	%Age	Primary studies
Ch1	Easy access of learning for students	19	46.34	PS3, PS17, PS33, PS2, PS37, PS17, PS19, PS12, PS40, PS10, PS15, PS25, PS23, PS16, PS27, PS11, PS1, PS5, PS8
Ch2	Redefining what teachers and schools mean to society	15	36.59	PS14, PS27, PS38, PS15, PS24, PS9, PS18, PS31, PS4, PS20, PS13, PS16, PS25, PS33, PS8
Ch3	Self-care, wellbeing and safe school opening	30	73.17	PS24, PS27, PS38, PS5, PS14, PS9, PS18, PS13, PS4, PS20, PS31, PS15, PS25, PS33, PS18, PS12, PS30, PS2, PS40, PS7, PS11, PS1, PS40, PS23, PS41, PS3, PS20, PS12, PS32, PS31
Ch4	Forging stronger links with parents and community	13	31.71	PS27, PS38, PS15, PS24, PS9, PS18, PS31, PS4, PS20, PS13, PS16, PS25, PS33
Ch5	Learning continuity and quality of education	22	53.66	PS24, PS27, PS38, PS5, PS14, PS9, PS18, PS13, PS4, PS20, PS31, PS15, PS25, PS33, PS12, PS2, PS11, PS1, PS23, PS41, PS3, PS20
Ch6	Ensuring distributive leadership	25	60.98	PS40, PS1, PS12, PS11, PS13, PS25, PS10, PS4, PS35, PS2, PS37, PS33, PS41, PS14, PS26, P28, PS13, PS39, PS36, PS7, PS6, PS32, PS30, PS3, PS15
Ch7	Emotional health of students and teachers	24	58.54	PS40, PS1, PS12, PS11, PS13, PS25, PS10, PS4, PS35, PS2, PS37, PS33, PS41, PS14, PS26, P28, PS13, PS39, PS36, PS7, PS6, PS32, PS30, PS4
Ch8	Exacerbated student attendance issues	16	39.02	PS7, PS12, PS27, PS23, PS15, PS24, PS9, PS18, PS31, PS33, PS37, PS41, PS1, PS40, PS26, PS3
Ch9	Engaging in constant updating	12	29.27	PS1, PS12, PS11, PS13, PS25, PS10, PS4, PS35, PS2, PS37, PS33, PS41
Ch10	Downplaying the threat and withholding bad news	18	43.90	PS2, PS40, PS13, PS15, PS16, PS23, PS41, PS33, PS34, PS11, PS24, PS26, PS27, PS29, PS31, PS36, PS37
Ch11	Communicating with transparency	11	26.83	PS15, PS16, PS23, PS41, PS33, PS34, PS11, PS24, PS26, PS27, PS29
Ch12	Effective communication methods	19	46.34	PS2, PS40, PS13, PS15, PS16, PS18, PS22, PS23, PS41, PS33, PS34, PS11, PS24, PS26, PS27, PS29, PS31, PS36, PS37
Ch13	Scheduling and other logistical challenges	9	21.95	PS12, PS32, PS31, PS7, PS6, PS10, PS19, PS39, PS37
Ch14	Equity gaps	27	65.85	PS24, PS27, PS38, PS5, PS14, PS9, PS18, PS13, PS4, PS20, PS31, PS15, PS25, PS33, PS18, PS12, PS30, PS2, PS40, PS7, PS11, PS1, PS40, PS23, PS41, PS3, PS20
Ch15	Striking a balance between technology and pedagogy	15	36.59	PS13, PS15, PS16, PS23, PS41, PS33, PS34, PS11, PS24, PS26, PS27, PS29, PS31, PS36, PS37
Ch16	Digital divides	28	68.29	PS38, PS5, PS14, PS9, PS18, PS13, PS4, PS20, PS31, PS15, PS25, PS33, PS18, PS12, PS30, PS2, PS40, PS7, PS11, PS1, PS40, PS23, PS41, PS3, PS20, PS12, PS32, PS31
Ch17	Technological needs to access teaching and learning	12	29.27	PS37, PS1, PS3, PS5, PS15, PS17, PS18, PS22, PS25, PS19, PS29, PS40
Ch18	The sudden transformation to online teaching	8	19.51	PS1, PS12, PS11, PS13, PS25, PS10, PS4, PS35
Ch19	Learning continuity and quality of education	17	41.46	PS2, PS40, PS13, PS15, PS16, PS23, PS41, PS33, PS34, PS11, PS24, PS26, PS27, PS29, PS31, PS36, PS37
Ch20	Assessment and evaluation challenges	11	26.83	PS37, PS1, PS3, PS5, PS15, PS17, PS18, PS22, PS25, PS19, PS29
Ch21	The problem of the practical course work	17	41.46	PS15, PS16, PS18, PS22, PS23, PS41, PS33, PS34, PS11, PS24, PS26, PS27, PS29, PS31, PS36, PS37, PS2, PS17, PS19
Ch22	Masking and ventilation mitigation strategies	19	46.34	PS2, PS4, PS15, PS16, PS18, PS22, PS23, PS41, PS33, PS34, PS11, PS24, PS26, PS27, PS29, PS31, PS36, PS37, PS2, PS17, PS19
Ch23	Cyber security of online education	23	56.10	PS40, PS1, PS12, PS11, PS13, PS25, PS10, PS4, PS35, PS2, PS37, PS33, PS41, PS14, PS26, P28, PS13, PS39, PS36, PS7, PS6, PS32, PS30
Ch24	Economic hardships of students and families	15	36.59	PS1, PS12, PS11, PS13, PS25, PS10, PS4, PS35, PS2, PS37, PS33, PS41, PS18, PS3, PS14

Ch3 (Self-care, Wellbeing, and Safe School Reopening) was underlined by 73.17% of the selected primary studies as the highly reported challenge. In countries where COVID-19 is still sweeping and the health system is overwhelmed, it is really hard to say when the safest time is to welcome students back in this troubled context. It concerns many that without student vaccinations, increases in student transmission are likely within schools, possibly contributing to COVID-19 resurgences in the community (Gurdasani et al., 2021). Any negligence or carelessness during this unpredictable pandemic will be paid with the student’s own life. When children’s education resumes physically and how it will be done specifically vary from school to school depending on the relax of COVID-19 in the locality. It could be also when the school community feels ready and confident about serving students face-to-face. Given any decision on full or partial school reopening, school leaders in collaboration with governments need to weigh the educational, public health, and socioeconomic benefits and risks in the local context.

Similarly, Ch16 (Digital Divides) was reported by 68.29% of the selected primary studies as another daunting challenge. Though it is the 4.0 era, school closures and the shift to distance learning have made the digital divides even more pronounced (Rogers and Sabarwal, 2020). Inevitably, digital transformation is synonymous with the advances of telecommunications infrastructure to a certain extent, which is closely related to the level of socioeconomic development of each locality. For many schools and students, even a stable Internet connection is a luxury. Accordingly, in low-income countries where the Internet is patchy and bandwidth is often low, access to online learning is significantly limited and only available for those in a privileged position (Mundy and Hares, 2020). While better-off teachers and students can quickly move to online lessons by seizing the advance of technology (Daniel, 2020), their peers from marginalized groups are far likely to suffer more and face more challenges (Mundy and Hares, 2020). Surely, many schools and students are falling behind in this technology race (Vlachopoulos, 2020).

Moreover, Ch14 (Equity Gaps, 65.85%) was indicated as a major challenge for smooth execution of school managerial activities during disruptive times. This pandemic has cut deep into the inequalities in access to education of marginalized students (Van Lancker and Parolin, 2020), regardless of whether their residencies are in a rich or poor country. The situation is even worse in school communities that are impoverished or with frequent armed conflicts, civil wars, or even ethnic divisions. The crisis is exacerbating pre-existing educational disparities, by depriving many of the most vulnerable young students of learning opportunities (Alasuutari, 2020; United Nations, 2020a). Due to the lack of schooling, vulnerable children are increasingly subjected to physical and psychological abuse, exploitation, and trafficking (Rogers and Sabarwal, 2020). The COVID-19 pandemic has been pushing back years of progress on gender equality (Fisher and Ryan, 2021). Worldwide, more than 1.5 billion students’ education is disrupted (World Bank Education, 2020; UNESCO, 2021a), almost half of these are female students. According to UNESCO estimates, millions of female students—at all levels of schooling—may not return to school during- and post-COVID-19 years (UNESCO, 2021b). This alarming number not only threatens the progress made on gender equality over the past many decades, but also puts girls globally at risk of child labor, victims of various forms of abuse and violence, forced marriage, and teenage pregnancy (Corlatean, 2020; United Nations, 2020a).

Further Ch6 (Ensuring Distributive Leadership) with the percentage of 60.98 was identified as a remarkable challenge for school leadership. Distributed leadership practices are vital in this time of crises (Gurr and Drysdale, 2020). Distributive leadership is the capability to initiate strategic transformation in an institution as demanded by the crisis situation. In a nutshell, leadership makes a transition from controlling to empowering others to mobilize collective strength and navigate different institutional challenges (Longmuir, 2021). Leaders must delegate some responsibility and authority to their followers so that leaders can focus more on work for things that tend to be strategic, and the organization can be agile to keep up with changes in the digital era (Astadi et al., 2022b). School leaders who were less likely to adopt a shared and collaborative approach have had to convert their leadership styles overnight to one of hasty decisions with slight or no team consultation, and correspond mandated decision, with a very short notice. It was not easy for principals to be prepared for and counter to the unprecedented and exceptional challenges.

Likewise, Ch7 (Emotional and Mental Health) with the frequency of 58.54% was found as another major difficulty. Mental health-related problems have been accelerated during the COVID-19 outbreaks (Adhanom Ghebreyeseus, 2020; Rogers and Sabarwal, 2020; WHO, 2020). The common mental-psychological state of school leaders, teachers, and students is confusion and anxiety. The risk of adverse mental-psychological states in school leaders is particularly high at most severe times of the pandemic (Argyropoulou et al., 2021). Many school leaders are facing pressures to prioritize the safety of the school community and provide learning continuity with limited resources, which puts them at high risk for depression, anxiety, burnout, and insomnia (De Matthews et al., 2021; Spyropoulou and Theodore Koutroukis, 2021).

The interruption of going to school, constant online teaching, and the pressure to make ends meet, let alone themselves or family members being infected with COVID-19, or the loss of loved ones increasingly deteriorate the mental and psychological health of the teachers (Baker et al., 2021; Gupta et al., 2021). Besides, school children are struggling with increased physical and mental health problems due to fear of COVID-19, home isolation, insufficient food and medicine, lack of parental supervision, and domestic violence (Rogers and Sabarwal, 2020; United Nations, 2020a). The stress or severe and persistent psychological trauma easily lead to many mental disorders post-COVID-19 (Fegert et al., 2020).

Also, Ch23 (Cyber Security of Online Education, 56.10%) was observed as the commonly quoted challenge for the principals (PS7, PS10, and PS12). Due to their greater reliance on technology and online interactions when working remotely, many schools are likely to fall prey to cybercriminals amid the pandemic (Kshetri, 2021). Recently, disruptive cyber security incidents are derailing virtual experiences of teaching and learning (Klein, 2020). Worse still, virtual incidents of abuse, harassment, and bullying of young students, let alone attacks against administrators and teachers, are increasing (Lobe et al., 2020; Henebery, 2021). However, many schools are inexperienced in tackling cyber attacks and putting in place steps to mitigate educational as well as social and emotional damage for students and teachers (Henebery, 2021). Such attacks targeting schools and young students, particularly during these turbulent times, are thoroughly reprehensible.

Additionally, Ch5 (Learning Continuity and Quality of Education) was reported as another significant challenge in the list with a frequency of 53.66%. First and foremost, school leaders have prioritized efforts to ensure learning continuity for students and teachers. Pressures come from the need to introduce a full range of appropriate learning for students with different backgrounds so as not to interrupt their learning. Accordingly, schools are encouraged to utilize a variety of online learning modalities (World Bank Education, 2020). Additionally, low- or non-technological approaches (i.e., broadcast lessons, learning packs, and printed materials for home delivery) should be ready for those with limited technology access (World Bank Education, 2020). When learning continuity is put in place, the public raise the issue of quality of education (Rogers and Sabarwal, 2020; Parveen et al., 2021). One thing is for sure, ensuring a holistic experience for students regardless of the form of teaching and learning during the complicated developments of the pandemic could give school leaders many sleepless nights.

While the universal awareness is that disruptive COVID-19 times are unprecedented, it is important that we consider some of the pressures school leaders are facing as they continue to go beyond the call of duty to support school-aged children and young people during a global health crisis. Whether there is a dearth of available research on the topic how school principals are responding to this pandemic situation, some emerging insights about school leadership during COVID-19 is available. A few intentions are offered for contemplation, in due course, about empirical propositions.

There are many studies regarding leadership and its challenges; however, there was a lack of literature that included the effect of pandemics that would reflect the same situation that the world is facing during COVID-19. Focusing on crises, such as COVID-19, the current study is relevant as no previous research has discussed issues where the entire educational system worldwide is heavily affected with many interruptions. Therefore, this paper brought light to various leadership challenges, and their possibility and criticality.

The findings of the study will not only contribute to the education sector to discuss the leadership problems of K-12 school principals in times of turbulence. The study may assist educational institutions in gaining a better understanding of the challenges for smooth running of management in disruptive times of COVID-19. Moreover, the study would provide fruitful results to raise management standards and to overcome the shortcomings of the education sector in this disruptive time. Ultimately, the results of the study will be of great benefit for the principals of public schools, researchers in school management, and leadership as well as education policy makers.

However, one of the drawbacks of the present study regarding systematic literature review is that there is much possibility for missing any previous literature. Therefore, the same applied process would provide different results and findings due to the unavailability of all libraries and repositories.

## Conclusion

The educational process worldwide has been heavily interrupted due to the COVID-19 pandemic. Accordingly, most educational institutions shifted from in-class education toward E-learning and suffered from again-and-again closure and uncertainty of continuity of learning. There is a universal call for significant increase in leadership role, responsibility, and resilience in educational institutions.

Educational institutions during COVID-19 face the unique challenges of smoothly maintaining the process of learning while ensuring that it is still beneficial. Therefore, educational leaders must understand what challenges are confronting them toward the smooth running of schools in this crucial time. The present study explored that the most-frequency-significance challenges influencing school activities during the COVID-19 pandemic were related to (1) self-care, wellbeing, and safe school opening; (2) learning continuity and quality of education; (3) ensuring distributive leadership; (4) emotional and mental health; (5) equity gaps; (6) digital divides; and (7) cyber security of online education. These finding should be seriously considered as the role of the principals is central in schools and it cannot be ignored.

## Implications

The study works as a contribution to K-12 school leadership by providing guidance for current and future leaders in crisis based on practical investigation, experiences, and recommendations. The findings of the current study are believed to have profound implications for future research. Critical challenges of leadership are substantial to be considered by school administration, policy makers, and future research. The identification of these leadership challenges helps fill the existing gaps in school leadership knowledge in the context of COVID-19. By addressing critical challenges facing school leaders in times of turbulence, school administration can revisit these results when navigating their own challenges. Policy makers can also leverage these findings to make necessary adjustments to school policy to better prepare current and would be school leaders for future crisis. These findings also upsurge the concern for further quantitative and/or qualitative investigation on school leadership challenges in disruptive times according to different contexts and the degree to which school leaders respond to a crisis. Besides, these leadership challenges are of significance for researchers to develop a crisis-response model for school leadership.

## Data Availability Statement

The original contributions presented in the study are included in the article/supplementary Material, and further inquiries can be directed to the corresponding author.

## Author Contributions

KP presented the main idea and wrote the first draft of the manuscript. PT and AA contributed to conduct the methodology. SA proofread the manuscript before submission. EN and TX helped in the revision of the manuscript. All authors contributed to the article and approved the submitted version.

## Conflict of Interest

The authors declare that the research was conducted in the absence of any commercial or financial relationships that could be construed as a potential conflict of interest.

## Publisher’s Note

All claims expressed in this article are solely those of the authors and do not necessarily represent those of their affiliated organizations, or those of the publisher, the editors and the reviewers. Any product that may be evaluated in this article, or claim that may be made by its manufacturer, is not guaranteed or endorsed by the publisher.

## Footnotes

^1^ https://www.tandfonline.com/

^2^ https://us.sagepub.com/en-us/nam/home

^3^ link.springer.com

^4^ https://www.emerald.com/insight/content/journals

^5^ www.wiley.com

^6^ https://www.academia.edu/

^7^ https://scholar.google.com
