# Supplementary material for: Identifying the Leadership Challenges of K-12 Public Schools During COVID-19 Disruption: A Systematic Literature Review
Source: Front Psychol. 2022 Mar 31;13:875646. doi: 10.3389/fpsyg.2022.875646 (PMC9009316; doi:10.3389/fpsyg.2022.875646)
Supplement: Supplementary file 1 [file Data_Sheet_1.docx]

**Appendix-A: List of selected primary studies along with Quality Evaluation score**

| **ID** | **Reference** | **Publication Year** | **Scopus / WoS IF** | **Database** | **QE1** | **QE2** | **QE3** | **QE4** | **QE5** | **T. Score** |
| --- | --- | --- | --- | --- | --- | --- | --- | --- | --- | --- |
| PS1 | Francom, G. M., Lee, S. J., & Pinkney, H. (2021). Technologies, challenges and needs of k-12 teachers in the transition to distance learning during the COVID-19 pandemic. *TechTrends*, *65*(4), 589-601. | 2021 | Q2 | Springer | 1.0 | 1.0 | 0.5 | 0.5 | 1.0 | 4.0 |
| PS2 | Alsaleh, A. (2021). Professional learning communities for educators’ capacity building during COVID-19: Kuwait educators’ successes and challenges. *International Journal of Leadership in Education*, 1-20. | 2021 | Q1 | Taylor & Francis | 1.0 | 0.5 | 1.0 | 1.0 | 0.5 | 4.0 |
| PS3 | Harris, A. (2020), "COVID-19 – school leadership in crisis?", Journal of Professional Capital and Community, Vol. 5 No. 3/4, pp. 321-326. https://doi.org/10.1108/JPCC-06-2020-0045 | 2020 | Q1 | Emerald | 1.0 | 0.5 | 1.0 | 1.0 | - | 3.5 |
| PS4 | Hulme, M., Beauchamp, G., Clarke, L., & Hamilton, L. (2021). Collaboration in times of crisis: Leading UK schools in the early stages of a pandemic. *Leadership and Policy in Schools*, 1-20. | 2021 | Q2 | Taylor & Francis | 1.0 | - | - | 1.0 | 1.0 | 3.0 |
| PS5 | Lucena Rodríguez, C., Mula-Falcón, J., Domingo Segovia, J., & Cruz-González, C. (2021). Fighting windmills: a female principal's story during COVID-19. *School Leadership & Management*, 1-18. | 2021 | Q1 | Taylor & Francis | 0.5 | 1.0 | 0.5 | 0.5 | 0.5 | 3.0 |
| PS6 | Huber, S. G., & Helm, C. (2020). COVID-19 and schooling: evaluation, assessment and accountability in times of crises—reacting quickly to explore key issues for policy, practice and research with the school barometer. Educational Assessment, Evaluation and Accountability, 32(2), 237-270. | 2020 | Q1 / IF 2 | Springer | 0.5 | 0.5 | 1.0 | 0.5 | - | 2.5 |
| PS7 | Reid, D. B. (2021). Suppressing and sharing: how school principals manage stress and anxiety during COVID-19. *School Leadership & Management*, 1-17. | 2021 | Q1 | Taylor & Francis | 0.5 | 1.0 | - | 1.0 | 1.0 | 3.5 |
| PS8 | Adams, D., Cheah, K. S., Thien, L. M., & Md Yusoff, N. N. (2021). Leading schools through the COVID-19 crisis in a South-East Asian country. *Management in Education*, 08920206211037738. | 2021 | Q2 | Sage Publications | 1.0 | - | 0.5 | 0.5 | 1.0 | 3.0 |
| PS9 | Doll, K., Ragan, M., Calnin, G., Mason, S., & House, K. (2021). Adapting and enduring: Lessons learned from international school educators during COVID-19. *Journal of Research in International Education*, *20*(2), 114-133. | 2021 | Q2 | Sage Publications | 1.0 | 1.0 | - | - | 1.0 | 3.0 |
| PS10 | Thornton, K. (2021). Leading through COVID-19: New Zealand secondary principals describe their reality. Educational Management Administration & Leadership, 49(3), 393-409. | 2021 | Q1 | Sage Publications | 0.5 | 1.0 | 1.0 | 0.5 | 0.5 | 3.5 |
| PS11 | Beauchamp, G., Hulme, M., Clarke, L., Hamilton, L., & Harvey, J. A. (2021). ‘People miss people’: A study of school leadership and management in the four nations of the United Kingdom in the early stage of the COVID-19 pandemic. Educational Management Administration & Leadership, 49(3), 375-392. | 2021 | Q1 | Sage Publications | - | 1.0 | 1.0 | 1.0 | 0.5 | 3.5 |
| PS12 | Kaden, U. (2020). COVID-19 school closure-related changes to the professional life of a K–12 teacher. *Education Sciences*, *10*(6), 165. | 2020 | Q2 | Google Scholar | 0.5 | 1.0 | 0.5 | 1.0 | 1.0 | 4.0 |
| PS13 | Jopling, M., & Harness, O. (2021). Does COVID-19 offer English school leaders the opportunity to rethink schools?. *Management in Education*, 0892020621994307. | 2021 | Q2 | Sage Publications | 0.5 | 0.5 | 1.0 | 1.0 | 0.5 | 3.5 |
| PS14 | Starr, J. P. (2020). On Leadership: Responding to COVID-19: Short-and long-term challenges. Phi Delta Kappan, 101(8), 60-61. | 2020 | Q2 | Sage Publications | 1.0 | 0.5 | 1.0 | 0.5 | 0.5 | 3.5 |
| PS15 | Chamberlain, L., Lacina, J., Bintz, W. P., Jimerson, J. B., Payne, K., & Zingale, R. (2020). Literacy in lockdown: Learning and teaching during COVID‐19 school closures. The Reading Teacher, 74(3), 243-253. | 2020 | IF: 1.632 | Wiley | 0.5 | 0.5 | 1.0 | 1.0 | 0.5 | 3.5 |
| PS16 | Williams, M. L., Morse, B. L., DeGraffenried, W., & McAuliffe, D. L. (2021). Addressing stress in high school students during the COVID-19 pandemic. NASN School Nurse, 36(4), 226-232. | 2021 | Q4 | Sage Publications | 0.5 | 1.0 | 1.0 | 1.0 | 1.0 | 4.5 |
| PS17 | Collie, R. J. (2021). COVID-19 and teachers’ somatic burden, stress, and emotional exhaustion: examining the role of principal leadership and workplace buoyancy. Aera Open, 7, 2332858420986187. | 2021 | IF: 2.299 | Sage Publications | 1.0 | 1.0 | 1.0 | - | 0.5 | 3.5 |
| PS18 | Alsaleh, A.A. (2021), "The roles of school principals and head teachers in mitigating potential learning loss in the online setting: calls for change", International Journal of Educational Management, Vol. 35 No. 7, pp. 1525-1537. https://doi.org/10.1108/IJEM-03-2021-0095 | 2021 | Q2 | Emerald | - | 1.0 | 1.0 | 0.5 | 0.5 | 3.0 |
| PS19 | Alinsunurin, J. (2020). School learning climate in the lens of parental involvement and school leadership: lessons for inclusiveness among public schools. *Smart Learning Environments*, *7*(1), 1-23. | 2020 | N/A | Springer | - | 1.0 | 1.0 | 1.0 | - | 3.0 |
| PS20 | Leung, M. W., Waters, J. L., & Ki, Y. (2021). Schools as spaces for in/exclusion of young Mainland Chinese students and families in Hong Kong. *Comparative Migration Studies*, *9*(1), 1-19. | 2021 | Q1 | Springer | 0.5 | 1.0 | 1.0 | 1.0 | 0.5 | 4.0 |
| PS21 | Netolicky, D. M. (2020). School leadership during a pandemic: navigating tensions. Journal of Professional Capital and Community, 5(3/4), 391-395. | 2020 | Q1 | Emerald | 1.0 | - | - | 1.0 | 1.0 | 3.0 |
| PS22 | Pittman, J., Severino, L., DeCarlo-Tecce, M. J., & Kiosoglous, C. (2021). An action research case study: Digital equity and educational inclusion during an emergent COVID-19 divide. *Journal for Multicultural Education*. | 2021 | Q1 | Emerald | 0.5 | 0.5 | 1.0 | 0.5 | 0.5 | 3.0 |
| PS23 | Shamir-Inbal, T., & Blau, I. (2021). Facilitating emergency remote K-12 teaching in computing-enhanced virtual learning environments during COVID-19 pandemic-blessing or curse?. *Journal of Educational Computing Research*, *59*(7), 1243-1271. | 2021 | Q1 / IF:3.008 | Sage Publications | 1.0 | 1.0 | - | - | 1.0 | 3.0 |
| PS24 | Nishan, F., & Mohamed, A. (2021). Emerging stronger: Policy directions for COVID-19 and beyond for public schools in the Maldives. *Fulbright Review of Economics and Policy*. | 2021 | N/A | Emerald | 0.5 | 1.0 | 0.5 | 1.0 | 1.0 | 4.0 |
| PS25 | Sadiq, A. A., Kapucu, N., & Hu, Q. (2020). Crisis leadership during COVID-19: the role of governors in the United States. *International Journal of Public Leadership*. | 2020 | N/A | Emerald | 0.5 | 1.0 | - | 1.0 | 1.0 | 3.5 |
| PS26 | Gyang, T. S. (2020). Educational leadership response to the COVID-19 pandemic crisis in Nigeria. *International Studies in Educational Administration*, *48*(3), 73-79. | 2020 | N/A | Emerald | 1.0 | 1.0 | 0.5 | 1.0 | 0.5 | 4.0 |
| PS27 | Dotan, S., Katzir, T., Lipka, O., & Shaul, S. (2021). Elementary School Literacy Teachers’ Perceptions and Challenges During COVID-19. Academia Letters. | 2021 | N/A | Academia | 1.0 | 1.0 | 1.0 | - | - | 3.0 |
| PS28 | Narayanan, V., Wokutch, R. E., Ghobadian, A., & O'Regan, N. (2021). Toward a strategic approach to studying COVID-19 pandemic. *Journal of Strategy and Management*. | 2021 | Q2 | Emerald | - | 1.0 | 1.0 | 0.5 | - | 2.5 |
| PS29 | Pollock, K. (2020). School Leaders’ Work During the COVID-19 Pandemic: A Two-Pronged Approach. *International Studies in Educational Administration*, *48*(3), 38. | 2020 | N/A | Emerald | 1.0 | 1.0 | 0.5 | 1.0 | 1.0 | 4.5 |
| PS30 | McLeod, S., & Dulsky, S. (2021). Resilience, reorientation, and reinvention: School leadership during the early months of the COVID-19 pandemic. In *Frontiers in Education* (p. 70). Frontiers. | 2021 | Q2 | Google Scholar | 0.5 | 1.0 | 0.5 | 0.5 | 1.0 | 3.5 |
| PS31 | O’Toole, C., & Simovska, V. (2021). Same storm, different boats! The impact of COVID-19 on the wellbeing of school communities. *Health Education*. | 2021 | Q3 | Emerald | 1.0 | 0.5 | 1.0 | 0.5 | 0.5 | 3.5 |
| PS32 | Rehm, M., Moukarzel, S., Daly, A. J., & Del Fresno, M. (2021). Exploring online social networks of school leaders in times of COVID‐19. British Journal of Educational Technology, 52(4), 1414-1433. | 2021 | Q1 | Wiley | 0.5 | - | 1.0 | 0.5 | 0.5 | 2.5 |
| PS33 | Fotheringham, P., Harriott, T., Healy, G., Arenge, G., & Wilson, E. (2021). Pressures and influences on school leaders navigating policy development during the COVID‐19 pandemic. British Educational Research Journal. | 2021 | Q1 | Taylor & Francis | 0.5 | 0.5 | 1.0 | 1.0 | 1.0 | 4.0 |
| PS34 | Leech, N. L., Gullett, S., Cummings, M. H., & Haug, C. (2020). Challenges of remote teaching for K-12 teachers during COVID-19. *Journal of Educational Leadership in Action*, *7*(1), 1. | 2020 | N/A | Google Scholar | - | 1.0 | 1.0 | 0.5 | 1.0 | 3.5 |
| PS35 | Pattison, K. L., Hoke, A. M., Schaefer, E. W., Alter, J., & Sekhar, D. L. (2021). National survey of school employees: COVID‐19, school reopening, and student wellness. Journal of School Health, 91(5), 376-383. | 2021 | Q1 | Wiley | 0.5 | 0.5 | 1.0 | 0.5 | 0.5 | 3.0 |
| PS36 | Aagaard, E. M., & Earnest, M. (2021). Educational leadership in the time of a pandemic: Lessons from two institutions. FASEB BioAdvances, 3(3), 182-188. | 2021 | N/A | Wiley | 1.0 | 0.5 | 0.5 | - | 0.5 | 2.5 |
| PS37 | Varela, D. G., & Fedynich, L. (2020). Leading schools from a social distance: Surveying south texas school district leadership during the COVID-19 pandemic. In *National Forum of Educational Administration and Supervision Journal* (Vol. 38, No. 4, pp. 1-10). | 2020 | N/A | Google Scholar | 0.5 | 0.5 | 1.0 | 1.0 | 1.0 | 4.0 |
| PS38 | Huck, C., & Zhang, J. (2021). Effects of the COVID-19 Pandemic on K-12 Education: A Systematic Literature Review. *New Waves-Educational Research and Development Journal*, *24*(1), 53-84. | 2021 | N/A | Google Scholar | - | 0.5 | 1.0 | 1.0 | 0.5 | 3.0 |
| PS39 | Zhang, T. (2021). Chinese parents’ perception of emergency remote K-12 teaching-learning in China during the COVID-19 pandemic. *Asian Journal of Distance Education*, *16*(1), 16. | 2021 | N/A | Google Scholar | 0.5 | 1.0 | 1.0 | 0.5 | 1.0 | 4.0 |
| PS40 | Harris, A., & Jones, M. (2020). COVID 19–school leadership in disruptive times. *School Leadership & Management*, *40*(4), 243-247. | 2020 | Q1 | Google Scholar | 1.0 | 0.5 | 1.0 | 1.0 | 1.0 | 4.5 |
| PS41 | Francisco, C. D., & Nuqui, A. V. (2020). Emergence of a Situational Leadership during COVID-19 Pandemic Called New Normal Leadership. Online Submission, 4(10), 15-19. | 2020 | N/A | Google Scholar | 1.0 | - | 1.0 | 1.0 | - | 3.0 |
