# Supplementary material for: Identifying the Leadership Challenges of K-12 Public Schools During COVID-19 Disruption: A Systematic Literature Review
Source: Front Psychol. 2022 Mar 31;13:875646. doi: 10.3389/fpsyg.2022.875646 (PMC9009316; doi:10.3389/fpsyg.2022.875646)
Supplement: Supplementary file 2 [file Data_Sheet_2.docx]

**Appendix-B: Kendall's Coefficient of Concordance (W)**

library(DescTools)

anxiety <- data.frame(rater1=c(3,3,3,4,3,4,2,3,3,2,2,2,3,4,2),

rater2=c(3,4,3,5,3,4,2,4,3,3,2,3,3,3,2),

rater3=c(3,3,4,4,3,4,1,3,3,3,1,2,3,3,2))

KendallW(anxiety, TRUE)

# with test results

KendallW(anxiety, TRUE, test=TRUE)

# example from Siegel and Castellan (1988)

d.att <- data.frame(

id = c(4,21,11),

airfare = c(5,1,4),

climate = c(6,7,5),

season = c(7,6,1),

people = c(1,2,3),

program = c(2,3,2),

publicity = c(4,5,7),

present = c(3,4,6),

interest = c(8,8,8)

)

KendallW(t(d.att[, -1]), test = TRUE)

# which is perfectly the same as

friedman.test(y=as.matrix(d.att[,-1]), groups = d.att$id)

Table: Kendall's coefficient of concordance test

| Data Set | Kendall Chi‐Squared | df | Subjects | Raters | *p* value | W |
| --- | --- | --- | --- | --- | --- | --- |
| CSOD | 35.434 | 14 | 15 | 3 | 0.001267 | 0.8436765 |
